# Facile Supermolecular Aptamer Inhibitors of L-Selectin

**DOI:** 10.1371/journal.pone.0123034

**Published:** 2015-03-31

**Authors:** Elizabeth K. Chang, Mark A. Eckert, M. Monsur Ali, Hamidreza Riazifar, Egest J. Pone, Linan Liu, Weian Zhao

**Affiliations:** Sue and Bill Gross Stem Cell Research Center, Chao Family Comprehensive Cancer Center, Edwards Lifesciences Center for Advanced Cardiovascular Technology, Edwards Lifesciences Center for Advanced Cardiovascular Technology, Department of Biomedical Engineering, and Department of Pharmaceutical Sciences, University of California Irvine, Irvine, California, 92697, United States of America; Beckman Research Institute of the City of Hope, UNITED STATES

## Abstract

Multivalent interactions occur frequently in nature, where they mediate high-affinity interactions between cells, proteins, or molecules. Here, we report on a method to generate multivalent aptamers (Multi-Aptamers) that target L-selectin function using rolling circle amplification (RCA). We find that the L-selectin Multi-Aptamers have increased affinity compared to the monovalent aptamer, are specific to L-selectin, and are capable of inhibiting interactions with endogenous ligands. In addition, the Multi-Aptamers efficiently inhibit L-selectin mediated dynamic adhesion in vitro and homing to secondary lymphoid tissues in vivo. Importantly, our method of generating multivalent materials using RCA avoids many of the challenges associated with current multivalent materials in that the Multi-Aptamers are high affinity, easily produced and modified, and biocompatible. We anticipate that the Multi-Aptamers can serve as a platform technology to modulate diverse cellular processes.

## Introduction

Nature often takes advantages of the principles of multivalency, in which many low affinity interactions lead to robust, high affinity interactions, to mediate contacts between proteins, molecules, and cells [1]. For example, during leukocyte homing, clusters of L-selectin on the surface of activated leukocytes effectively interact with multiple low-affinity carbohydrate ligands to effect enhanced functional binding affinity (avidity) [2]. Recently, researchers have begun to take advantage of the principles of multivalency to engineer systems with high avidity to modulate normal and disease biology. In fact, L-selectin itself has been a popular target for novel multivalent materials, with examples that highlight both the potential and limitations of multivalent materials for modulation of biology [3–13].

Due to L-selectin’s essential roles in leucocyte trafficking in inflammation and injury, inhibition of L-selectin mediated leucocyte rolling has potential applications in anti-inflammatory medication [14]. Several groups have developed multivalent materials to modulate L-selectin mediated rolling via inhibitors that either promote L-selectin shedding or block it from binding to endogenous ligands. Such inhibitors include cross-linked antibodies [3], bivalent DNA aptamers [4–6], and synthetic multivalent ligands [7–13]. Crosslinked antibodies (bivalent antibodies or antibody saturated beads) have shown potential to modulate signaling events downstream of L-selectin clustering more effectively than their monovalent antibody counterparts [3]. Although monovalent DNA aptamers are specific for L-selectin are capable of blocking L-selectin mediated interactions with endothelial cells, both in vitro and in vivo [4–6], bivalent aptamers have increased affinity for surface L-selectin with more potent blocking abilities [6]. Finally, synthetic multivalent ligands, including neoglycopolymers and tetravalent sialyl Lewis X (SLeX) molecules, mimic endogenous ligands of L-selectin and lead to robust inhibition of L-selectin function [7–12]. Interestingly, in addition to their blocking function, some of these multivalent materials can induce metalloproteinase-dependent shedding of L-selectin [11,13,15]. Mowery et al hypothesize that L-selectin shedding occurs selectively in response to synthetic multivalent compounds with high ligand density whereas multivalent compounds with lower ligand densities lead instead to blocking of L-selectin function [13].

Unfortunately, several challenges may prevent ready translation of these novel modulators of L-selectin function: antibodies are costly and may elicit adverse immune response in vivo [16], synthetic multivalent ligands require extensive and complex chemistries that are not easily modified [13,15], and DNA aptamers require high effective dosages in order to inhibit L-selectin activity [4–6]. Therefore, our group aims to develop a multivalent biomaterial that is biocompatible, reproducible and modifiable, that will more effectively inhibit L-selectin activity at lower dosages for future in vivo use.

**Fig 1:**
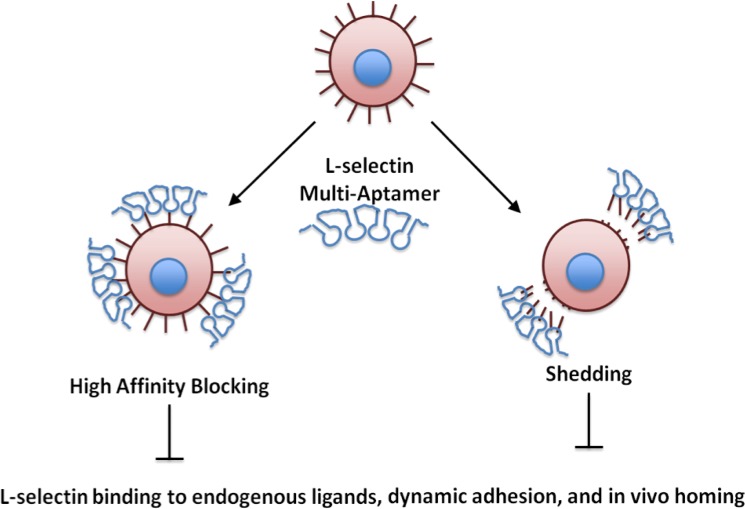
Potential mechanisms of action of multivalent interactions with L-selectin. We hypothesize that the Multi-Aptamer may either inhibit L-selectin function via high affinity, multivalent binding to L-selectin or by inducing shedding of surface L-selectin via multivalent interactions.

We have previously utilized a simple isothermal enzymatic reaction called rolling circle amplification (RCA) to generate multivalent scaffolds to capture rare cells and deliver chemotherapeutic agents [17–20]. In RCA reactions, a DNA polymerase such as phi29 polymerase extends a primer by replicating from a circular DNA template many times to generate a long, single-stranded DNA (ssDNA) molecule [21–26]. The RCA product consists of repetitive sequence elements that are complementary to the circular template that can be easily modified by varying the circular template sequence. Here, we propose to harness the versatility of RCA to generate long, multivalent ssDNA sequences that incorporate an L-selectin aptamer (LS-Multi-Aptamer). We hypothesize that the multiplicity of the DNA aptamers will increase the avidity for L-selectin and therefore more effectively and efficiently modulate its function in vitro and in vivo. This may include both more effective inhibition of L-selectin binding to endogenous ligands, or induction of clustering and shedding of L-selectin from the surface of the cell (**Fig 1**) [13]. Our Multi-Aptamer platform possesses several key advantages, including that it is easily reproduced and can be modified by simply adjusting the parameters of the reaction: 1) by modifying the template sequence, the Multi-Aptamer could target multiple ligands simultaneously, 2) by adjusting the reaction time, the Multi-Aptamer’s length and overall valency could be controlled, and 3) by incorporating modifications, such as fluorophores, the Multi-Aptamer could be used as a potential tool in diagnostics. Although here we demonstrate the potential of the Multi-Aptamer in the context of modulation of L-selectin function, we believe it will have utility as a platform technology to target other signaling pathways.

## Materials and Methods

### 1. Materials

All DNA sequences used in this study were purchased from Integrated DNA Technologies, Inc. (Coralville, IA). Materials (FITC-dUTPs, dNTPs, T4 PNK, phi29 polymerase, T4 ligase, and buffers) used for rolling circle amplification were purchased from Thermo Scientific (Waltham, MA). 100K centrifugal devices to purify Multi-Aptamer products were purchased from Pall Life Sciences (Port Washington, NY). Jurkat cells were obtained from ATCC and cultured following manufacturer’s protocol; human brain endothelial cells were from Cell Systems and cultured following manufacturer’s protocol. RPMI-1650 was obtained from Gibco and fetal bovine serum (FBS) from Atlantic Biologicals. Cyclosporin A and phorbol myristate acetate (PMA) were purchased from Sigma Aldrich (St. Louis, MO); recombinant TNFα was from R&D Systems (Minneapolis, MN). The non-blocking anti-L-selectin antibody (clone 4G8), and the enzyme-linked immunosorbent assay (ELISA) for detection of soluble L-selectin were purchased from R&D Systems. The FITC-labeled anti-L-selectin blocking antibody (DREG56) and the antibodies (N-18 anti-L-selectin and HRP-conjugated secondary antibodies) used for Western blots were purchased from Santa Cruz Biotechnology, Inc. (Dallas, TX). Penicillin-streptomycin, SYBR Safe, and FITC annexin V cell apoptosis kit were purchased from Life Technologies (Carlsbad, CA).

### 2. Rolling circle amplification

Circular template sequences (**Table 1**) were ligated via T4 DNA ligase following phosphorylation by T4 polynucleotide kinase (T4 PNK) as previously described [20]. The circular templates were purified by ethanol precipitation and polyacrylamide gel electrophoresis (PAGE). Purified circular templates were mixed with primers on an equimolar basis, dNTPs, and phi29 polymerase to complete the RCA reaction. RCA was carried out for 10 minutes at 30°C and products verified by agarose gel electrophoresis (BioRad ChemiDoc XRS+). The RCA products were purified using 100K centrifugal devices. FITC labeled RCA products were synthesized by incorporating a 1:10 ratio of FITC-labeled dUTPs to dNTPs in the reaction mixture. Incorporation of FITC-dUTP was verified via gel electrophoresis imaging.

**Table 1:** DNA sequences used in this study.

**Name**	**Sequence (5’→3’)**
LS Circle Template	ACCTTGTACTGGTTACCTTGGCTAAAAAAAAAAAAAAAAAAAAAGTAAGCCGAAGCCATTACGTTTAGC
LS Primer	CCAGTACAAGGTGCTAAACGTAAT
SC Circle Template	TGGGATACTGAACGGATGCCTTACAAAAAAAAAAAAAAAAAAAAACCTTATAGTTGTTGATTCCACCCG
SC Primer	TTCAGTATCCCACGGGTGGAATCA
LS Aptamer	TAGCCAAGGTAACCAGTACAAGGTGCTAAACGTAATGGCTTCGGCTTAC
SC Aptamer	AGAGGCTACAGCGATAAGTCGACATTGCTGACCGTACCTAGTAATACGT

### 3. Multi-Aptamer affinity and competitive binding assays

For determination of aptamer and Multi-Aptamer binding affinities, we incubated 200,000 Jurkat cells with a range of concentrations of FITC-labeled monovalent aptamer (100 pM, 1 nM, 10 nM, 100 nM, 1 μM, 100 μM, 500 μM) or FITC-labeled LS-Multi-Aptamer (10 pM, 100 pM, 1 nM, 10 nM, 100 nM, 200 nM, 500 nM). Cells were fixed and mean fluorescence determined via flow cytometry. All values were normalized to the maximum fluorescence intensity detected for the monovalent aptamer and the Multi-Aptamer. For competitive binding experiments, 1 million Jurkat cells were treated simultaneously with 100 nM FITC-labeled blocking antibody (DREG56) and increasing concentrations of LS-Multi-Aptamer and controls, ranging from 0.2 nM to 100 nM for Multi-Aptamers and 0.2 nM to 1 μM for monovalent aptamers for 35 minutes at 4°C. The cells were washed twice in PBS, fixed in 2% paraformaldehyde (PFA) for 15 minutes, and resuspended in 400 μL of PBS for flow cytometry analysis with a BD LSR II. The IC50 were obtained using GraphPad Prism. For confocal analysis, Jurkat cells were treated identically to above with 100 nM FITC-labeled LS-Multi-Aptamer, but pre-labeled by staining with 1 μM Cell Tracker Red for 15 minutes at 37°C. Imaging was performed on an Olympus FV10i confocal microscope.

### 4. Cell viability and apoptosis

1 million Jurkat cells were untreated or treated with 100 nM LS-Multi-Aptamers or RCA products containing scrambled sequences (SC-Multi-Aptamers) or 50 μM cyclosporin A for 1 or 6 hours at 37°C and stained with FITC-labeled annexin V and propidium iodide per the manufacturer’s instructions (Life Technologies; Carlsbad, CA). Annexin V and PI positive cells were quantified by flow cytometery with a BD LSR II. To determine cell viability, 300,000 Jurkat cells were grown for 1, 6, or 24 hours in the presence of 100 nM SC- or LS-Multi-Aptamer before addition of XTT reagents per manufacturer’s protocol (Biotium; Hayward, CA). Untreated cells and cells treated with 50 μM cyclosporin A served as controls. Absorbance at 450–500 nm was measured with a Biotek Synergy HT plate reader.

### 5. ELISA

1 million Jurkat cells were untreated or treated with 100 nM LS- or SC-Multi-Aptamer and 100 ng/ml PMA for 30 minutes at 37°C. The cells were centrifuged at 400 x g for 5 minutes and the supernatants were collected for ELISA per the manufacturers recommended protocol (R&D Systems). Absorbance at 450 nm was measured with a Biotek Synergy HT plate reader and used to derive a concentration via four parameter logistic nonlinear regression to a standard curve.

### 6. Western blot

1 million Jurkat cells were untreated or treated with 100 nM LS- or SC-Multi-Aptamer and 200 ng/ml PMA for 30 minutes at 37°C. Cells were centrifuged at 400 x g for 5 minutes and the cell pellets were collected. After washing cell pellets in PBS, the cells were lysed in 50 μL of 1x radioimmunoprecipitation assay (RIPA) buffer. Cells were incubated on ice for 10 minutes and mixed by vortexing before centrifugation. 20 μg of total protein was loaded into each well of an 8–12% SDS-PAGE gel and subjected to electrophoresis. Samples were then transferred to a PVDF membrane, blocked with 5% milk in TBS-T for 1 hour, and probed with 1:200 anti-L-selectin goat polyclonal antibody (N-18) overnight at 4°C. The PVDF membrane was probed for 1 hour with 1:5000 HRP conjugated donkey anti-goat antibody. The bands were visualized by ECL reagents and chemiluminescence imaging with a BioRad ChemiDoc XRS+.

### 7. SLeX binding assay

200,000 Jurkat cells were untreated or incubated with 100 nM of SC- or LS-Multi-Aptamer or the L-selectin or control monovalent aptamer (LS- and SC-Aptamer, respectively) for 30 minutes prior to incubation with 50 μg/ml of FITC-labeled SLeX (Glycotech; Gaithersburg, MD) for 1 hour in PBS with 1% human serum albumin. Cells were washed with PBS, fixed with PFA, and fluorescence assessed via flow cytometry and normalized to untreated cells.

### 8. Dynamic adhesion assay

Human brain endothelial cells (ECs) were grown to confluency in a 35 mm dish 24 hours before the assay. ECs were activated with 50 ng/ml TNFα for 6 hours prior to adhesion assay. 10^5^ Jurkat cells were untreated or treated with 100 nM LS- or SC-Multi-Aptamer or 100 nM LS- and SC-Aptamer for 30 minutes at 37°C while simultaneously labeled with 1 μM Cell Tracker Green CMFDA. Jurkat cells were washed with PBS, then added to the plate containing ECs while rotating at 60 rpm as previously described [27]. Following a 5 minute incubation, media and non-adherent Jurkat cells were carefully decanted and the EC plate fixed with 4% PFA. Plate was washed with PBS and adhered cells imaged with Nikon Eclipse Ti fluorescence microscope and quantified as the number of fluorescently-labeled Jurkat cells per unit area.

### 9. *In vivo* homing

Female non-obese diabetic, severe combined immunodeficiency gamma (NOD SCID gamma/NSG) mice were obtained from Jackson Laboratory. 15 million Jurkat cells were treated with 100 nM L-selectin or scrambled monovalent aptamers (i.e., LS- or SC-Aptamer) or LS- or SC-Multi-Aptamer for 30 minutes at 37°C. Cells were washed in PBS prior to retro-orbital injection into female NSG mice. Mice were sacrificed after two hours and mesentary lymph nodes collected via blunt dissection. Whole genomic DNA was isolated using the DNeasy Tissue kit (Qiagen) and the relative proportion of human Jurkat cells in the mouse tissue quantified with PCR-based detection of human Alu sequences and mouse GAPDH genomic DNA sequences. The following primers were used: hAlu-F: 5’-CACCTGTAATCCCAGCACTTT-3’; hAlu-R: 5’-CCCAGGCTGGAGTGCAGT-3’; msGAPDH-F: 5’-GCACAGTCAAGGCCGAGAAT-3’; msGAPDH-R: 5’-GCCTTCTCCATGGTGGTGAA-3’. PCR conditions were: 95°C for 2 minutes; 40 cycles at 95°C for 30 seconds, 65°C for 20 seconds, and 72°C for 20 seconds using The Alu sequence was normalized against the relative quantity of GAPDH for each respective control as ΔCt = Ct^Alu^-Ct^GAPDH^ and expressed as 2^ΔCt^ to establish a unitless homing index for each treatment [28]. All animal experiments were performed with permission of the University of California, Irvine’s Institutional Animal Care and Use Committee (IACUC# 2012–3068) following the National Institute of Health Guide for the Care and Use of Laboratory Animals.

## Results and Discussion

### 1. Design and synthesis of L-selectin Multi-Aptamers

**Fig 2:**
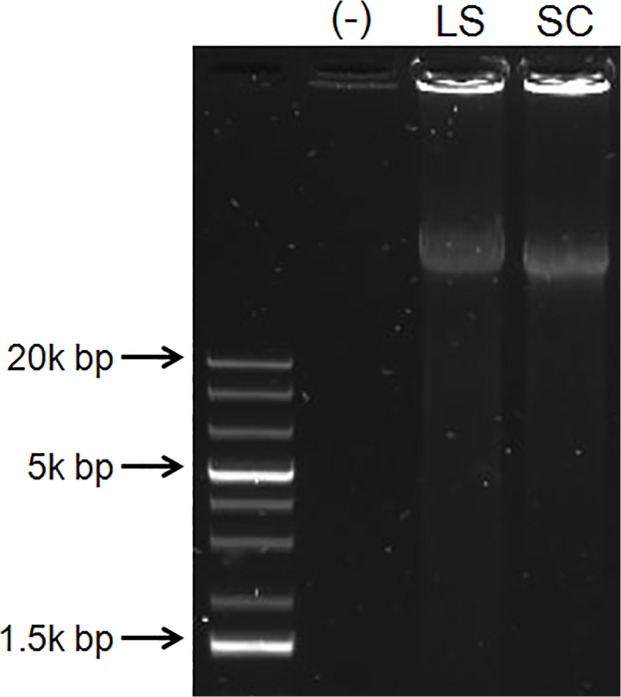
Synthesis of the Multi-Aptamers. Following a 10 minute RCA reaction, the LS- and SC-Multi-Aptamer DNA products were only generated in the presence of the primer (LS, SC). The negative sample (-) does not contain a primer.

Monovalent L-selectin aptamers recognize and bind to L-selectin positive cells and are capable of blocking L-selectin function in vivo [4–6]. In particular, Hicke and coworkers first identified L-selectin binding DNA aptamers and demonstrated their utility in inhibiting lymphocyte adhesion and trafficking in vitro and in vivo [5]. In this study, we generated multivalent forms of these identified aptamer sequences to specifically bind to cell surface L-selectin using RCA with phi29 polymerase (**Fig 1**). Briefly, the circular template consists of the complementary sequence of the L-selectin aptamer (hereafter, abbreviated LS) or a scrambled sequence (hereafter, abbreviated SC). Aptamer units are separated by a 20 nucleotide poly(T) sequence in the RCA product (reflected by the poly(A) sequence in the circle template). The resulting long, linear ssDNA product incorporated multiple copies of the L-selectin aptamer, or the scrambled sequence (LS-Multi-Aptamer and SC-Multi-Aptamer, respectively). We verified synthesis of the LS- and SC-Multi-Aptamers via agarose gel electrophoresis (**Fig 2**). Only in the presence of the primer was RCA of the circular template completed. RCA reactions of 10 minutes at 30°C consistently yielded Multi-Aptamers of high molecular weights. In our previous studies using gel electrophoresis to examine hybridization between RCA product with short complementary strands at different molar ratios [29,30] and using atomic force microscopy [31]. We found RCA products generated under this condition would correspond to a valency of approximately 30. It should be noted that this system is remarkably flexible: the identity of the aptamer, distance between aptamer units, and valency can all be adjusted by varying the sequences utilized or the reaction conditions.

### 2. LS-Multi-Aptamers specifically bind to L-selectin

**Fig 3:**
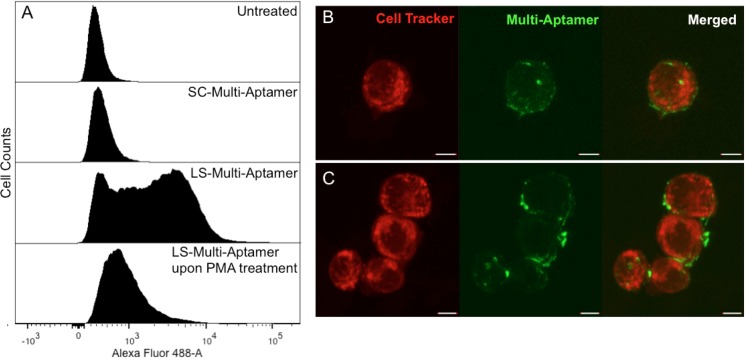
LS-Multi-Aptamer specifically binds to L-selectin on Jurkat cells. (A) Flow cytometry histograms of Jurkat cells stained with the indicated Multi-Aptamers and controls. (B) Confocal microscope images demonstrate that FITC-labeled LS-Multi-Aptamer (green) effectively binds to Jurkat cells (red). (C) Confocal microscope images demonstrate that cross-linking of multiple Jurkat cells by the LS-Multi-Aptamer can be occasionally found. Scale bar is 5 μm.

To evaluate binding of the LS-Multi-Aptamers to cell surface L-selectin, we utilized T lymphocyte Jurkat cells which express high levels of surface L-selectin as a model system. These cells display cell behavior similar to primary leukocytes, including coordinated tethering, rolling, and adhesion to activated endothelial cells as well as in vivo recruitment to secondary lymphoid tissues [32,33]. FITC-labeled RCA products were generated by adding FITC-dUTP to the RCA reaction in a 1:10 ratio to unlabeled dNTPs (S1 **Fig**). Jurkat cells were untreated or treated with FITC-labeled LS-Multi-Aptamer, or FITC-labeled SC-Multi-Aptamer and analyzed via flow cytometry. Jurkat cells treated with phorbol myristate acetate (PMA) subsequently stained with LS-Multi-Aptamer served as a control (**Fig 3A**). PMA rapidly activates protein kinase C (PKC) dependent L-selectin shedding through zinc-dependent metalloproteinases [34]. The flow cytometry analysis indicated that the LS-Multi-Aptamer specifically bound to cell surface L-selectin: in the absence of L-selectin, via PMA-induced shedding, the FITC-labeled multi-aptamer could not be detected on the cell surface.

To qualitatively investigate the binding properties of the LS-Multi-Aptamer for Jurkat cells, we performed confocal microscopy on Jurkat cells treated with FITC-labeled LS-Multi-Aptamers. Jurkat cells were stained with Cell Tracker Red CMTPX and simultaneously incubated with FITC-labeled LS-Multi-Aptamer. Confocal images were taken immediately after the cells were fixed. The FITC-labeled LS-Multi-Aptamer effectively enveloped the surface of the Jurkat cells (**Fig 3B**), which was confirmed with z-stack image reconconstruction (S2 **Fig**). In some cases, the FITC-labeled Multi-Aptamer cross-linked multiple Jurkat cells (**Fig 3C**), which further demonstrates the multivalent effect of Multi-Aptamers. Recognizing that the reaction conditions including especially the concentrations of RCA products and immune cells would likely be very different between in vitro and in vivo settings, it is interesting to note that any potential RCA-mediated cell aggregation in blood vessels in vivo may pose a desirable effect in inhibiting inflammatory cell trafficking or a detrimental effect due to the potential formation of embolism, which we will study in future work.

### 3. LS-Multi-Aptamers have increased affinity for L-selectin compared to their monovalent counterparts

**Fig 4:**
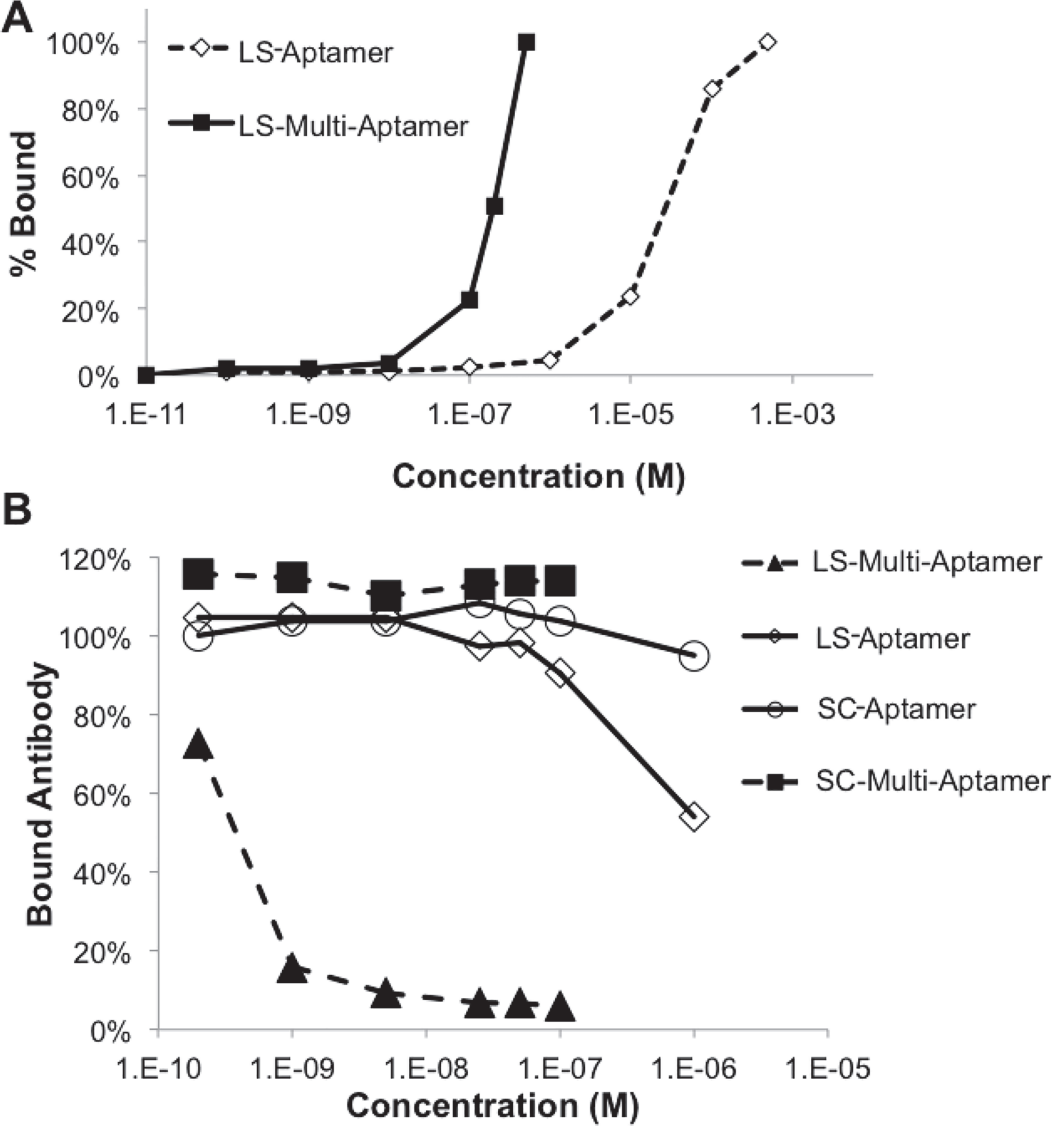
LS-Multi-Aptamer has a higher binding affinity for L-selectin than the monovalent aptamer. (A) Jurkat cells were incubated with fluorescent monovalent L-selectin aptamer (LS-Aptamer) or the LS-Multi-Aptamer at the indicated concentrations and fluorescence assessed with flow cytometry. (B) Jurkat cells were simultaneously treated with FITC-labeled blocking antibody, DREG56 (100 nM) and increasing concentrations of the indicated reagents. The mean fluorescence for each sample is normalized to the mean fluorescence of the untreated sample labeled with FITC-DREG56.

We predicted that the cooperative interactions of numerous aptamer moieties would lead to the LS-Multi-Aptamer having a higher affinity for L-selectin than the monovalent aptamer (i.e., LS-Aptamer). To test this hypothesis, we incubated Jurkat cells with a range of concentrations of FITC-labeled LS-Aptamer and FITC-labeled LS-Multi-Aptamer (10^-10^-10^-4^ M). Fluorescence was assessed via flow cytometry and normalized to the maximum fluorescence labeling achieved. Increasing the multivalency of the aptamers increased the binding affinity for cell-surface L-selectin: the LS-Multi-Aptamer had an approximately 10^3^ higher apparent affinity for L-selectin than the monovalent aptamer (**Fig 4A**). We note that the incorporated FITC via dUTP might interfere the binding between Multi-Aptamer and target molecules although it, if any, did not appear to be significant based on our data. A future alternative using radiolabeled Multi-Aptamer can circumvent this potential issue.

To validate our observations regarding the affinity of the LS-Multi-Aptamer, we also performed a competition assay in which increasing concentrations (10^-10^-10^-5^ M) of the LS-Multi-Aptamers or the LS-Aptamer were co-incubated with the L-selectin antibody DREG56 that competitively binds to the same epitope of L-selectin as the L-selectin aptamer [6]. Compared to the LS-Aptamer, SC-Aptamer, and the SC-Multi-Aptamer, the LS-Multi-Aptamer out-competed the antibody at lower concentrations (**Fig 4B**). At nanomolar concentrations, the LS-Multi-Aptamer blocked the FITC-labeled antibody from binding to cell surface L-selectin, whereas the monovalent form was outcompeted by the antibody at these concentrations. The LS-Aptamer failed to block the FITC-labeled antibody until its concentration was 10^4^ times higher than that of the LS-Multi-Aptamer. Specifically, the IC50 values are ~0.75 nM and >1 μM for LS-Multi-Aptamer and LS-Aptamer, respectively (**Fig 4B**). This corresponds to 22.5 nM inhibition potential per aptamer unit (given that each RCA product contains approximately 30 repeating units (see Section 3.1)) which is significantly higher than that of LS-Aptamer (IC50 of >1 μM). This data suggests that the multivalent form interacts more strongly with cells than does the monovalent form—by having multiple cooperative binding interactions, unbinding at a single site does not release the multi-aptamer, and re-binding of that site is likely to occur. In addition, the steric effect of long Multi-Aptamer, once bound to even a single L-selectin on the cell surface, might facilitate the inhibition of antibody binding, therefore contribute to the largely decreased IC50 of Multi-Aptamer compared to LS-Aptamer.

### 4. LS-Multi-Aptamers do not affect viability of target cells in vitro

**Fig 5:**
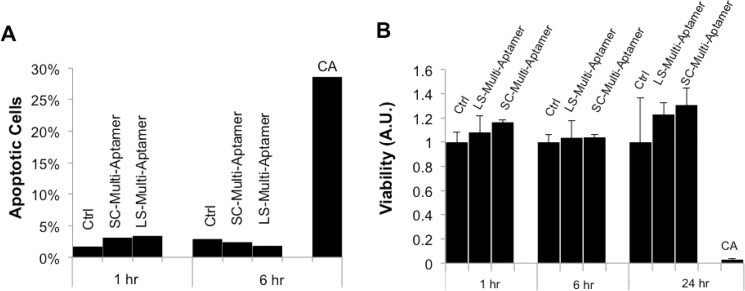
The Multi-Aptamer does not induce apoptosis or affect cell viability. (A) Jurkat cells were untreated or treated with SC-Multi-Aptamer, LS-Multi-Aptamer. Treatment with cyclosporine A (CA) served as a control. Apoptosis was assessed at the indicated time points by flow cytometry analysis of annexin V and propidium iodide (PI). (B) To assess potential effects on cell viability or proliferation, Jurkat cells were treated with the indicated compounds. Cell viability was assessed by introduction of XTT reagent. Treatment with 50 μM CA served as a control. Error bars are standard error of the mean (SEM).

To address potential concerns of biocompatibility of the Multi-Aptamers, we performed apoptosis and viability assays on Jurkat cells with the LS-Multi-Aptamers at various time points. For 1-hour and 6-hour time points, the frequency of PI and Annexin V negative cells remained over 95% for cells treated with SC- and LS-Multi-Aptamers (**Fig 5A**). As a positive control, Jurkat cells were treated with cyclosporin A (50 μM) for 6 hours to induce apoptosis [35]. To validate the apoptosis results and assess potential effects on cell proliferation, we performed cell viability assays using a XTT assay which assays cell viability via colorimetric measurement of cellular respiration. Even at 24 hours, there was no significant difference in cell viability in cells treated with the Multi-Aptamers (**Fig 5B**). These results indicate that the LS-Multi-Aptamer does not induce significant off-target toxicities or affect cell viability, making it an appealing compound for in vivo use.

### 5. LS-Multi-Aptamers block binding to endogenous ligands without inducing shedding

**Fig 6:**
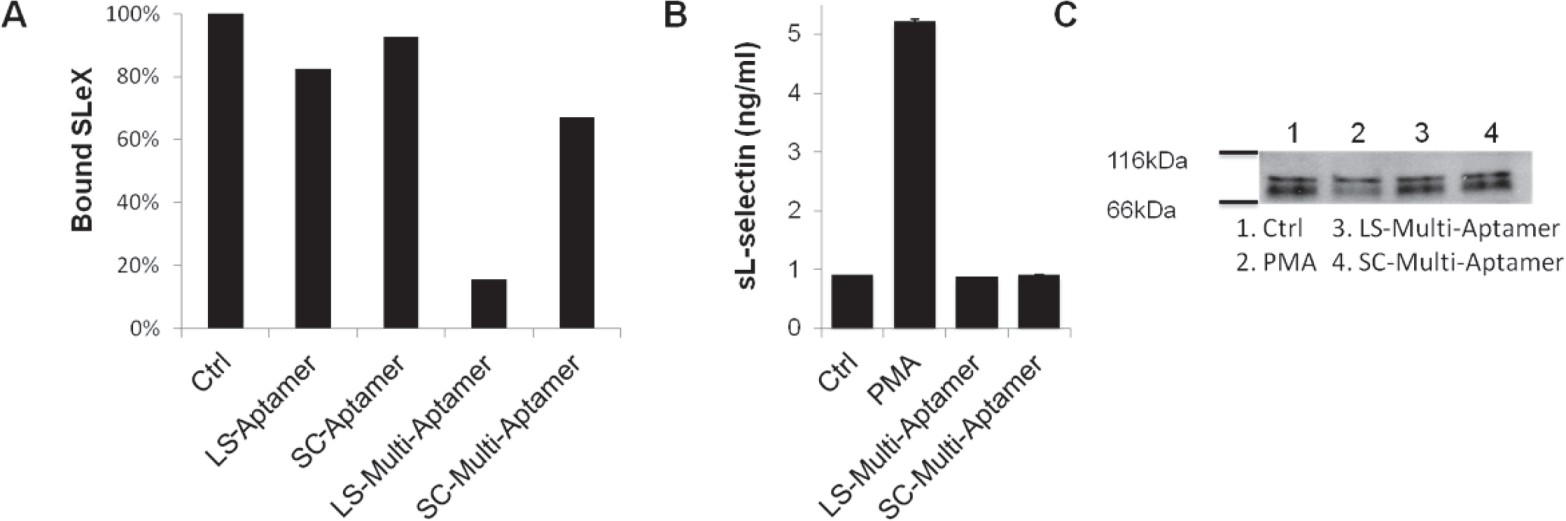
LS-Multi-Aptamer blocks binding to endogenous ligands without inducing shedding. (A) Relative percentage of bound SLeX following incubation of Jurkat cells with the indicated monovalent aptamers and Multi-Aptamers. (B) ELISA quantification of shed L-selectin following treatment of Jurkat cells with the indicated Multi-Aptamers or PMA. (C) Western blot analysis of Jurkat cell-associated L-selectin following the indicated treatments. Error bars are SEM.

Due to the strong affinities of the Multi-Aptamer for L-selectin, we next explored its potential to modulate L-selectin function by inhibiting binding to endogenous ligands. One of the most well-established ligands for L-selectin is SLeX, which directly interacts with L-selectin and plays key roles in mediating initial tethering and rolling of leukocytes [36]. To test this hypothesis, we incubated 200,000 Jurkat cells with 100 nM of SC- or LS-Multi-Aptamer or the SC- or LS-Aptamer for 30 minutes prior to incubation with 50 μg/ml of FITC-labeled SLeX for 1 hour. Fluorescence was assessed via flow cytometry and normalized to untreated cells. Despite some off-target inhibition of Jurkat cell-SLeX binding in the presence of the SC-Multi-Aptamer, the LS-Multi-Aptamer still led to a dramatic reduction in Jurkat cell interaction with SLeX that was much more robust than inhibition with monovalent aptamers (**Fig 6A**). This data suggests that the LS-Multi-Aptamer can inhibit binding to endogenous ligands, either through blocking or inducing shedding of L-selectin from the cell surface.

As previous studies have reported that multivalent polymers and cross-linked antibodies induce L-selectin shedding [3,13], we next investigated if the LS-Multi-Aptamer led to shedding of L-selectin. After Jurkat cells were untreated or treated with PMA, LS-Multi-Aptamer, and SC-Multi-Aptamer, we collected the supernatants for analysis by ELISA to detect soluble L-selectin (**Fig 6B**) and lysed the treated cells for analysis of cell-associated L-selectin by Western blot (**Fig 6C**). PMA, a well-established inducer of L-selectin shedding, induced L-selectin shedding while the untreated, LS-Multi-Aptamer, and SC-Multi-Aptamer treated cells retained their cell surface L-selectin. Our results therefore suggest that the LS-Multi-Aptamer does not induce shedding of L-selectin but rather blocks its functional binding to endogenous ligands (**Fig 6A–6C**). Although previous studies have reported that multivalent L-selectin ligands induced shedding, shedding induced by synthetic polyvalent L-selectin ligands was modest in comparison to PMA treatment [13], which raises the question of if L-selectin shedding is necessary for modulation of function. It should also be noted that studies of L-selectin monovalent and bivalent aptamers have not demonstrated shedding [4–6]. Interestingly, synthetic multivalent carbohydrate ligands only induced shedding of L-selectin at millimolar concentrations while blocking L-selectin function at micromolar concentrations [9]. It has been hypothesized that shedding of L-selectin by multivalent ligands depends on both the number of ligands as well as the distance between the ligands [9]. In the future, we plan to tailor the parameters of the LS-Multi-Aptamer to directly determine if the physical properties of the Multi-Aptamers can be optimized to mediate shedding versus blocking. For example, the length of the Multi-Aptamer can be adjusted by varying the duration of the RCA reaction and the distance between aptamers can be modified by adjusting the length of the poly(T) spacer.

### 6. LS-Multi-Aptamers modulate L-selectin mediated dynamic adhesion

**Fig 7:**
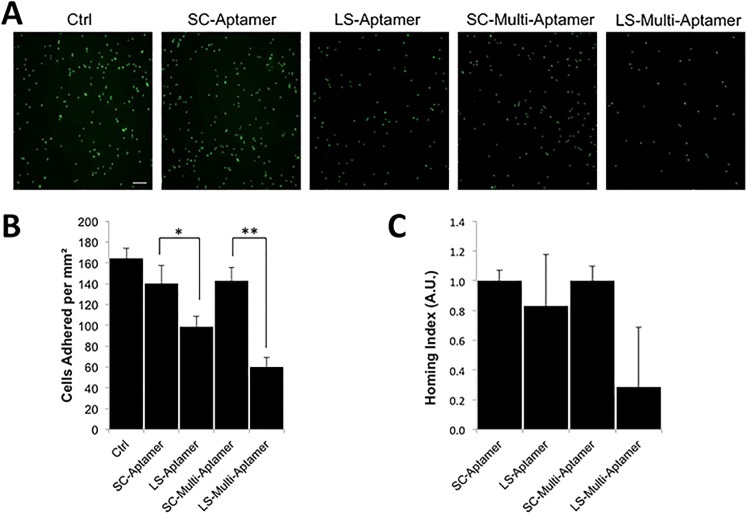
LS-Multi-Aptamer inhibits dynamic adhesion and homing in vitro and in vivo. (A) Representative images of Jurkat cells after dynamic adhesion to activated endothelial cells and treatment with the indicated aptamers or Multi-Aptamers. Scale bar is 20 μm. (B) Quantification of Jurkat cells that dynamically adhered to activated endothelial cells following the indicated treatments. * p < 0.05; ** p < 0.01. (C) Quantification of relative recruitment of Jurkat cells to the mesenteric lymph nodes following treatment with the monovalent SC- or LS-aptamer or SC- or LS-Multi-Aptamer, normalized to the respective control. Error bars are SEM.

After determining that the LS-Multi-Aptamer efficiently and specifically bound to surface L-selectin and inhibited interactions with endogenous ligands, we next attempted to determine if the LS-Multi-Aptamer inhibited the function of L-selectin. As L-selectin has well-characterized roles in mediating adhesion to endothelial cells, we tested this hypothesis by performing a dynamic adhesion assay. Briefly, Jurkat cells were labeled with Cell Tracker Green and untreated or treated with SC- or LS-Multi-Aptamer or monovalent aptamers before incubation on TNFα-activated human endothelial cells under shear stress to mimic conditions experienced by leukocytes in the peripheral vasculature [27]. After 5 minutes, the number of cells that had adhered under dynamic conditions was assayed via fluorescence microscopy. We found that the LS-Multi-Aptamer not only inhibited Jurkat cell binding to endothelial cells, but did so more effectively than the corresponding monovalent aptamer (**Fig 7A and 7B**).

### 7. LS-Multi-Aptamers inhibit in vivo homing

As our previous experiments suggested that the LS-Multi-Aptamer had clear potential to modulate L-selectin function, we next sought to determine its efficacy in vivo. Previous studies reported that the L-selectin aptamer was capable of blocking human T-cell homing to lymph nodes in vivo [5]. We therefore chose a model of T-cell homing to secondary lymphoid tissues to test the efficacy of the LS-Multi-Aptamer. Jurkat cells, like T-cells, will home specifically to secondary lymphoid tissues including mesenteric lymph nodes associated with the small bowel [33]. Briefly, 15 million Jurkat cells were untreated or treated with 100 nM SC- or LS-Aptamers or 100 nM SC- or LS-Multi-Aptamers before retro-orbital injection into NSG mice. Note that based on our in vitro antibody competition study (**Fig 4B**), under this condition, Multi-Aptamer would block >90% of the L-selectin binding sites on Jurkat cells. However, we did not necessarily aim for saturated Multi-Aptamer binding or associated with cells prior animal administration because we try to mimic the cell-drug interaction scenario in the circulation in vivo. 2 hours later, mice were sacrificed and the mesenteric lymph nodes collected. Total genomic DNA was isolated from the mesentery lymph nodes. The presence of human Jurkat cells in the sample was quantified by real-time PCR analysis for human-specific Alu sequences normalized to mouse GAPDH; each treatment group was normalized to the respective SC-Aptamer or Multi-Aptamer [28]. Treatment with the monovalent LS-Aptamer led to a modest trend toward reduced lymph node homing that did not reach significance. However, we found that the LS-Multi-Aptamer led to a robust trend toward decreased Jurkat cell recruitment to secondary lymphoid tissues that neared significance (p = 0.134) (**Fig 7C**).

Combined with our previous in vitro data, this indicates that the LS-Multi-Aptamer has potential as a novel modulator of L-selectin signaling as both a research tool and potential therapeutic. Many receptor signaling events are mediated by dimerization or higher-order interactions, which could be similarly modified with appropriate aptamers [37,38]. In addition, as L-selectin has established roles in trauma, systemic inflammatory syndromes, and sepsis, we are very interested in exploring the potential of the Multi-Aptamer in relevant animal models [14]. This will also entail a rigorous investigation of the pharmacokinetics, pharmacodynamics, and biodistribution of the Multi-Aptamers in vivo, especially in the context of mouse models of inflammation. In the future, we anticipate that the Multi-Aptamer system may be used as a platform technology to both modulate cell surface signaling and selectively deliver therapeutics to target cells.

## Conclusions

We have developed a multivalent aptamer system that binds with high avidity and specificity to human L-selectin. In vitro, the multivalent aptamer blocks L-selectin interactions with endogenous ligands and endothelial cells and binds specifically to L-selectin with 10^3^ fold higher affinity than monovalent L-selectin aptamers. In vivo, the multivalent aptamer shows promise of blocking homing to secondary lymphoid tissues at nanomolar concentrations. The biocompatibility and affinity of the Multi-Aptamer system make it a promising candidate for novel anti-inflammatory therapeutics or drug-delivery. We anticipate that our Multi-Aptamer technique can serve as a platform technology to increase aptamer avidity for in vivo applications as well as to modify other signaling pathways relevant to human health and disease.

## Supporting Information

S1 Fig Synthesis of FITC-labeled Multi-Aptamers. Following RCA reaction with 1:10 dilution of FITC-dUTP to dNTP, the RCA products were analyzed via gel electrophoresis. The panel on the left is stained with Sybr Safe, while in the panel on the right the fluorescent RCA product can be visualized in the absence of Sybr Safe staining.
(TIF)

S2 Fig LS-Multi-Aptamers interact with Jurkat cells. In this z-stack (planes indicated by yellow lines), the RCA product can be observed almost entirely enveloping the Jurkat cell, which is labeled with Cell Tracker Red. Scale bar is 5 μm.
(TIF)
