# Supplementary figures and images for: Facile Supermolecular Aptamer Inhibitors of L-Selectin
Source: PLoS One. 2015 Mar 31;10(3):e0123034. doi: 10.1371/journal.pone.0123034 (PMC4380364; doi:10.1371/journal.pone.0123034)

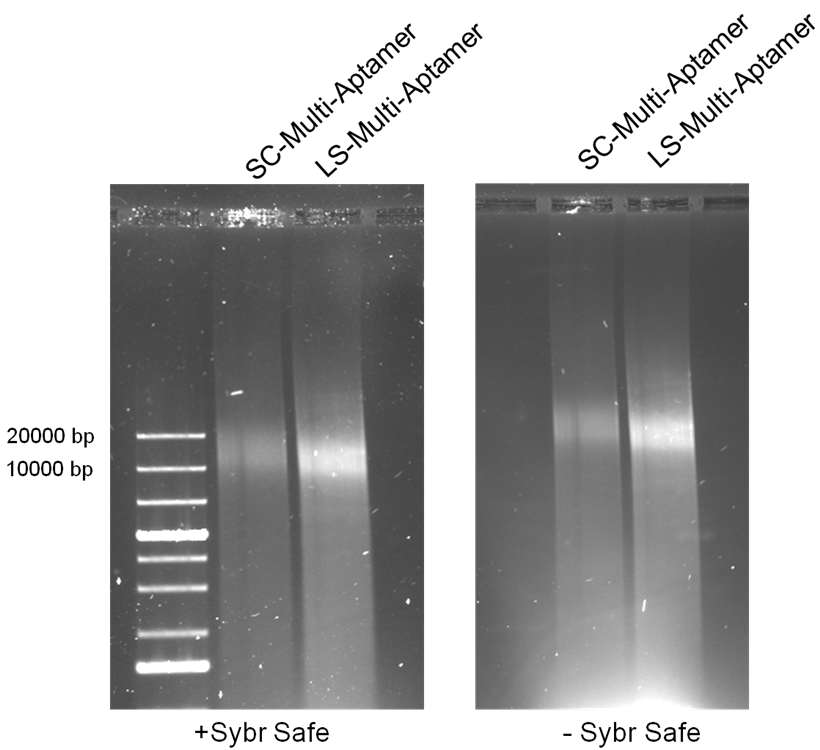

Supplement: S1 Fig — Following RCA reaction with 1:10 dilution of FITC-dUTP to dNTP, the RCA products were analyzed via gel electrophoresis. The panel on the left is stained with Sybr Safe, while in the panel on the right the fluorescent RCA product can be visualized in the absence of Sybr Safe staining. (TIF) [file pone.0123034.s001.tif]

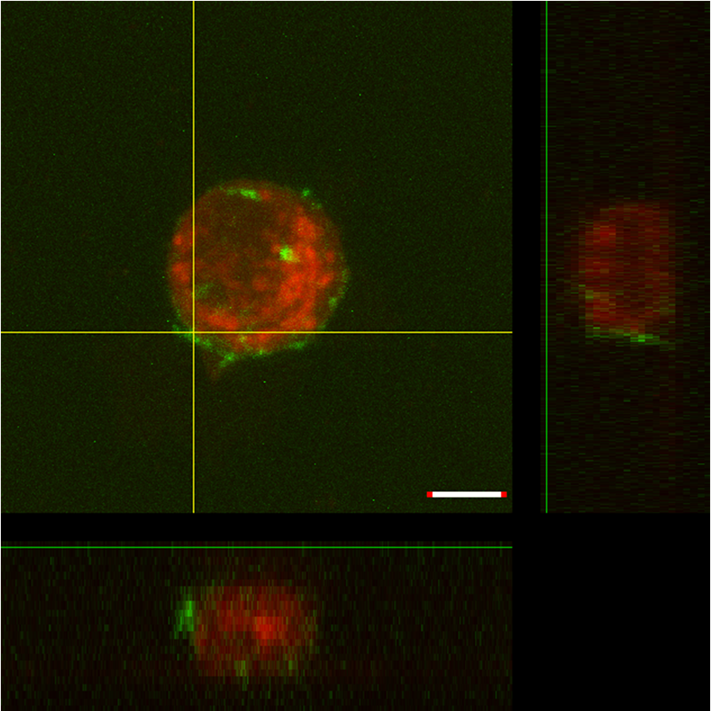

Supplement: S2 Fig — In this z-stack (planes indicated by yellow lines), the RCA product can be observed almost entirely enveloping the Jurkat cell, which is labeled with Cell Tracker Red. Scale bar is 5 μm. (TIF) [file pone.0123034.s002.tif]
